# Antibiotic Susceptibility of Biofilm Cells and Molecular Characterisation of *Staphylococcus hominis* Isolates from Blood

**DOI:** 10.1371/journal.pone.0144684

**Published:** 2015-12-14

**Authors:** Soraya Mendoza-Olazarán, Rayo Morfín-Otero, Licet Villarreal-Treviño, Eduardo Rodríguez-Noriega, Jorge Llaca-Díaz, Adrián Camacho-Ortiz, Gloria M. González, Néstor Casillas-Vega, Elvira Garza-González

**Affiliations:** 1 Servicio de Gastroenterología, Hospital Universitario Dr. José Eleuterio González, Universidad Autónoma de Nuevo León, Monterrey, Nuevo León, México; 2 Hospital Civil de Guadalajara, Fray Antonio Alcalde, and Instituto de Patología Infecciosa y Experimental, Centro Universitario de Ciencias de la Salud, Universidad de Guadalajara, Guadalajara, Jalisco, México; 3 Departamento de Microbiología e Inmunología, Facultad de Ciencias Biológicas, Universidad Autónoma de Nuevo León, Monterrey, Nuevo León, México; 4 Departamento de Patología Clínica, Hospital Universitario Dr. José Eleuterio González, Universidad Autónoma de Nuevo León, Monterrey, Nuevo León, México; 5 Servicio de Infectología, Hospital Universitario Dr. José Eleuterio González, Universidad Autónoma de Nuevo León, Monterrey, Nuevo León, México; 6 Departamento de Microbiología, Facultad de Medicina, Universidad Autónoma de Nuevo León, Monterrey, Nuevo León, México; University of Liverpool, UNITED KINGDOM

## Abstract

**Objectives:**

We aimed to characterise the staphylococcal cassette chromosome *mec* (SCC*mec*) type, genetic relatedness, biofilm formation and composition, *icaADBC* genes detection, *icaD* expression, and antibiotic susceptibility of planktonic and biofilm cells of *Staphylococcus hominis* isolates from blood.

**Methods:**

The study included 67 *S*. *hominis* blood isolates. Methicillin resistance was evaluated with the cefoxitin disk test. *mecA* gene and SCC*mec* were detected by multiplex PCR. Genetic relatedness was determined by pulsed-field gel electrophoresis. Biofilm formation and composition were evaluated by staining with crystal violet and by detachment assay, respectively; and the biofilm index (BI) was determined. Detection and expression of *icaADBC* genes were performed by multiplex PCR and real-time PCR, respectively. Antibiotic susceptibilities of planktonic cells (minimum inhibitory concentration, MIC) and biofilm cells (minimum biofilm eradication concentration, MBEC) were determined by the broth dilution method.

**Results:**

Eighty-five percent (57/67) of isolates were methicillin resistant and *mecA* positive. Of the *mecA*-positive isolates, 66.7% (38/57) carried a new putative SCC*mec* type. Four clones were detected, with two to five isolates each. Among all isolates, 91% (61/67) were categorised as strong biofilm producers. Biofilm biomass composition was heterogeneous (polysaccharides, proteins and DNA). All isolates presented the *icaD* gene, and 6.66% (1/15) isolates expressed *icaD*. This isolate presented the five genes of *ica* operon. Higher BI and MBEC values than the MIC values were observed for amikacin, vancomycin, linezolid, oxacillin, ciprofloxacin, and chloramphenicol.

**Conclusions:**

*S*. *hominis* isolates were highly resistant to methicillin and other antimicrobials. Most of the detected SCC*mec* types were different than those described for *S*. *aureus*. Isolates indicated low clonality. The results indicate that *S*. *hominis* is a strong biofilm producer with an extracellular matrix with similar composition of proteins, DNA and *N*-acetylglucosamine; and presents high frequency and low expression of *icaD* gene. Biofilm production is associated with increased antibiotic resistance.

## Introduction

*Staphylococcus hominis*, a coagulase-negative staphylococcus (CoNS) species, is an opportunistic pathogen that is one of the three most common isolates found in the blood of neonates and immunosuppressed patients.[1–3] In recent years, reports of *S*. *hominis* infection-induced bacteraemia, septicaemia, endophthalmitis, and endocarditis have increased in frequency.[2–7] *S*. *hominis* infections are often highly resistant to antibiotics and thus, are difficult to treat. Resistance to linezolid and vancomycin has been reported in several isolates [8–10]. Furthermore, methicillin resistance, which is associated with the *mecA* gene, has been found in up to 80% *S*. *hominis* isolates [11–13]. The *mecA* gene resides within a mobile genetic element called the staphylococcal cassette chromosome *mec* (SCC*mec*)[14] that was first described in *Staphylococcus aureus*. This element is related to *mec* gene complex classes A–E and *ccr* gene complex classes 1–8. Eleven types of SCC*mec* have been described (http://www.sccmec.org/Pages/SCC_TypesEN.html); however, *S*. *hominis* is prone to carry novel SCC*mec* types because of the presence of high-frequency non-typeable and new combinations of *mec* and *ccr* gene complexes.[11, 13, 15]

Nosocomial infections by CoNS are primarily associated with the use of medical devices, likely because of biofilm formation [16–18]. A biofilm is a community of bacteria living in an organised structure as cellular clusters or microcolonies. The biofilm is encapsulated in a matrix composed of an extracellular polymeric substance that is separated by open water channels. The water channels act as a primitive circulatory system to deliver nutrients and remove metabolic waste products. The biofilm allows bacteria to adhere to inert materials and to experience increased antibiotic resistance.[19, 20] Several CoNS species are more resistant to antibiotics when in a biofilm than when they exist as free-swimming planktonic cells. Therefore, because they were designed for planktonic cells, antibiotic treatments based on the protocols provided by the Clinical and Laboratory Standards Institute (CLSI) may fail to clear biofilm-related CoNS infections.[21]

A recent study described *S*. *hominis* biofilm production, their architecture and *icaADBC* frequency. [22] However, compared to other CoNS species, *S*. *hominis* is not categorised as a strong biofilm producer. Moreover, little information is available regarding the antibiotic susceptibility of *S*. *hominis* cells in a biofilm. Therefore, we aimed to characterise the SCC*mec* type, genetic relatedness, and ability to form biofilms for 67 clinical isolates of *S*. *hominis* obtained from blood cultures. The antibiotic susceptibilities of planktonic and biofilm cells of these strains were also compared.

## Materials and Methods

### Ethics statement

This study was performed with approval from the Ethics Committee of the School of Medicine of the Universidad Autónoma de Nuevo León (approval no. GA14-009). Because patient information was anonymized, only microbiological data were analysed. Therefore, the local ethics committee did not require informed consent.

### Clinical isolates

From January 2006 to December 2014, a total of 67 *S*. *hominis* clinical isolates from blood cultures were collected from two hospitals in Mexico: Hospital Civil Fray Antonio Alcalde (Guadalajara, Jalisco) and Hospital Universitario Dr José Eleuterio González (Monterrey, Nuevo León). All isolates were causative agents of laboratory-confirmed bloodstream infection according to criteria of the US Centers for Disease Control (http://www.cdc.gov/nhsn/pdfs/pscmanual/17pscnosinfdef_current.pdf). Isolates were kept at -70°C in *Brucella* broth containing 15% glycerol. Only one isolate per patient was used in the study.

### Isolate identification

Isolates were identified at the species level by API Staph Galleries (bioMérieux, Inc., Durham, NC, USA), according to the manufacturer’s instructions. Species identification was confirmed by partial sequencing of the 16S rRNA, as previously described.[23] Sequencing was performed at the Instituto de Biotecnología, Universidad Nacional Autónoma de México. DNA sequences were compared to genes in the US National Center for Biotechnology Information (NCBI) GenBank by using the BLAST algorithm (http://www.ncbi.nlm.nih.gov/BLAST).

### Methicillin resistance, SCC*mec* typing, and genetic relatedness

Methicillin resistance was evaluated by the cefoxitin disk test. The *mecA* gene was detected by polymerase chain reaction (PCR).[24] In the cefoxitin disk assay, results under 24 mm indicated resistant isolates, and results of 25 mm or greater indicated susceptible isolates.[25]

Identification of the SCC*mec* element type, according to the *ccr* class (*AB1*, *AB2*, *AB3*, or *C*) and *mec* class (A, B, or C), was performed as previously described.[24, 26] The *ccrAB4* type was determined by the method used by Oliveira *et al*.[27] with the modifications proposed by Zhang *et al*.[12] All SCC*mec* typing experiments were performed in duplicate. SCC*mec* was considered ‘non-typeable’ when the *ccr* and/or the *mec* complex did not amplify by PCR with any of the primer pairs. SCC*mec* was classified as ‘new’ when isolates contained a different combination of *ccr* and *mec* complexes as those previously reported for *S*. *aureus* by the International Working Group on the Classification of Staphylococcal Cassette Chromosome Elements (http://www.sccmec.org/).

Pulsed-field gel electrophoresis was performed as described for *S*. *aureus* [28], including modifications in the restriction enzyme and running conditions as described by Bouchami *et al*.[11] Specifically, isolate samples were digested with *Xho*I, and bands were separated by a CHEF-DRIII instrument (Bio-Rad Laboratories). Banding patterns were analysed visually by counting the bands using Labworks 4.5 software with 1% tolerance and 0.5 optimization settings. Similarity coefficients were generated from a similarity matrix, which was calculated with the Jaccard coefficient in the SPSS 22.0 software package. To define a clone two criterion previously described were used: a similarity cut-off of 80% [29] and a difference of ≤ six bands [30].

### Phenotypic biofilm assay

Semi-quantitative determination of biofilm formation was performed in duplicate by crystal violet staining as previously described [18, 31], with modifications to the normalisation approaches that compensate for growth rate differences [32, 33]. All isolates were tested in quadruplicate in two independent experiments. Polystyrene, 96-well, flat-bottom, non-treated plates with a low-evaporation lid were used for this assay.

The cut-offs proposed by Christensen *et al*. were used to classify the level of biofilm production [18]. Isolates with an OD of 0.25 or greater were considered to be strong biofilm producers, whereas isolates with ODs between 0.12 and 0.24 were considered to be weak biofilm producers. For quantitative analysis, the biofilm index (BI) was determined. For each experiment, the OD_600_ measurement for all cells (biofilm + planktonic cells OD_600_) was divided by the mean biofilm OD_570_ measurement of three wells per isolate: BI = total cells OD_600_ / biofilm density OD_570_. *Staphylococcus saprophyticus* ATCC 15305 (biofilm producer and *ica* operon-negative), *Staphylococcus epidermidis* ATCC 35984 (biofilm producer and *ica* operon-positive and *S*. *hominis* ATCC 27844 (biofilm non-producer and *ica* operon-negative) were used as control organisms.

### Biofilm detachment assays

Detachment assays were performed using sodium *meta*-periodate (NaIO4) to degrade β-1,6-linked polysaccharides, proteinase K to degrade proteins and DNase I to degrade DNA as described previously. [16] Briefly, each mature biofilms cultivated in tryptic soy broth with glucose 1% (TSB_glu1%_) were washed three times with PBS and were treated with (a) 40 mM NaIO_4_ in double-distilled H_2_O, (b) 0.1 mg/mL proteinase K (BIO-37037; Bioline) in 20 mM Tris-HCl (pH 7.5) with 100 mM NaCl or (c) 0.5 mg/ml DNase I (DN25; Sigma) in 5 mM MgCl_2_ for 24 h at 37°C. After, the biofilms were stained with crystal violet as described above. [18] Three wells containing uninoculated TSB_glu1%_ served as sterility control son each plate; the OD of these wells was used as spectrophotometric blanks. For each parallel run, the highest and the lowest OD values were removed to exclude outliers, and the remaining values were averaged. Percent detachment was calculated by the average difference between the treated wells and the untreated wells. The detachment results were classified as no detachment (<10%), intermediate detachment (10 to 50%), moderately strong (51–75%), and strong detachment (>75%) All isolates were tested in three parallel runs *Staphylococcus epidermidis* ATCC 35984 was included as a control (PIA as most abundant component).

### *icaADBC* operon detection and expression of the *ica*D

The *icaA*, *icaD*, *icaB*, *icaC* and *icaR* genes were detected by multiplex PCR.[34]. The *icaD* gene expression was detected in 15 selected isolates. *Staphylococcus hominis* cells were cultured in the same conditions used for biofilm formation (TSB_1%_ at 37°C) and harvested at mid-log phase (16 h). RNA was extracted using the High Pure RNA Isolation Kit (Roche, CA, USA) following the manufacturer’s instructions. The cDNA synthesis was performed using Transcriptor First Strand cDNA Synthesis Kit (Roche, CA, USA). Real-time PCR was performed using iQ SYBR Green Supermix with the primers previously reported [34] and the PCR conditions reported by Ciu *et al* [35]. *tuf* gene was used as internal control for normalization gene for expression of *icaD*. Amplification of *icaD* and *tuf* genes was detected by the presence of products of Tm of 81.12 and 72.14 in the melting curve, respectively. Amplification was confirmed by the presence of the corresponding PCR products on 2% agarose gel. *icaD* gene was considered as up-regulated if there was a relative change in expression higher than two (2^1^) than the *tuf* gene.

### Minimal inhibitory concentration (MIC)

Susceptibility testing was performed by using the broth microdilution method, as recommended by the CLSI.[25] The tested antibiotics included erythromycin, trimethoprim, amikacin, vancomycin, linezolid, oxacillin, ciprofloxacin, and chloramphenicol (Sigma Aldrich, Toluca, Mexico).

### Minimum biofilm eradication concentration (MBEC)

The assay reported by Ceri, H *et al*.[36] was used to determine the antibiotic susceptibility of biofilm cells. Bacterial biofilms were grown on polystyrene pegs in the Calgary Biofilm Device by utilising a microtitre plate (the MBEC^™^-Physiology and Genetics assay, Innovotech, Edmonton, AB, Canada), and following the manufacturer’s instructions. To begin, a bacterial inoculum of 1.0 McFarland and diluted 1:50 (~ 10^8^ CFU/mL) into TSB_glu1%_. To establish biofilm, 100 μL of the inoculum was added to each well of a 96-well microtiter plate. The peg lid was then fitted inside of this and the assembled device was placed on a gyrorotary shaker at ~150 revolutions per minute (rpm) in a humidified incubator for 18–24 h at 35°C. After incubation, pegs lid were rinsed twice with 100 μL PBS per peg to remove non-adherent cells and transfer the peg lid a new microtiter plate with 100 μL of twofold serial dilutions of antibiotics ranging from 1024 μg/mL to 0.06 μg/mL, in Müeller-Hinton broth (MHB) and MHB 1% NaCl (for oxacillin). Microtiter plates were then incubated at 35°C for 18 or 24 h, depending on the antibiotics tested. After antibiotic exposure, the peg lid was removed and rinsed twice with PBS and the biofilm disrupted by sonication for 8 s at 10% of the maximum amplitude (Branson 5800 Ultrasonic Cleaner) into MHB (recovery plate). The recovery plate was incubated for 24 h at 35°C. It was visually checked for turbidity in the wells and the MBEC was defined as minimum concentration of antimicrobial that eradicates the biofilm. Clear wells are evidence of eradication.

For the data analysis, it was considered as a difference significant when the isolates showed an increase > 2 fold for amikacin, ciprofloxacin, erythromycin, linezolid, oxacillin and trimethoprim, and an increase of > 3 fold for chloramphenicol in MBEC compared to MIC. This was established according at acceptable range of MIC for antibiotic quality control for *Staphylococcus aureus* ATCC 25913.

The MIC and MBEC were measured on three different occasions. In the case of non-concordance of the results, a fourth test was performed

### Statistical analysis

Analysis of variance (ANOVA) tests with the post-hoc Sidak correction were used to compare differences between MBEC, MIC, and BI values and averages (SPSS 20.0 software). A *p*-value of 0.05 or less was considered statistically significant.

## Results

### Methicillin resistance, SCC*mec* typing, and genetic relatedness

The cefoxitin disk test revealed that 85% (57/67) of isolates were resistant to methicillin and that all of these isolates tested positive for the *mecA* gene. Of the 57 *mecA*-positive isolates, 66.7% (38/57) had a new SCC*mec* complex. Of these isolates carrying new SSC*mec* complexes, 32 amplified *mec* complex A and *ccrAB1* (New1), four isolates carried *mec* complex A, *ccrAB1*, and *ccrC* (New2), one isolate had *mec* complex A and *ccrC* (New3), and one isolate had *mec* complex A, *ccrAB4*, and *ccrC* (New4). Isolates with non-typeable SCC*mec* complexes represented 24.6% of the total (14/57). Of these, ten isolates had only *mec* class A (NT1) and four isolates did not have *ccr* or *mec* (NT2). Only 8.8% (5/57) of isolates carried a typeable SCC*mec* complex: three had type I, one had type III, and one had type VI SCC*mec* complexes.

**Fig 1:**
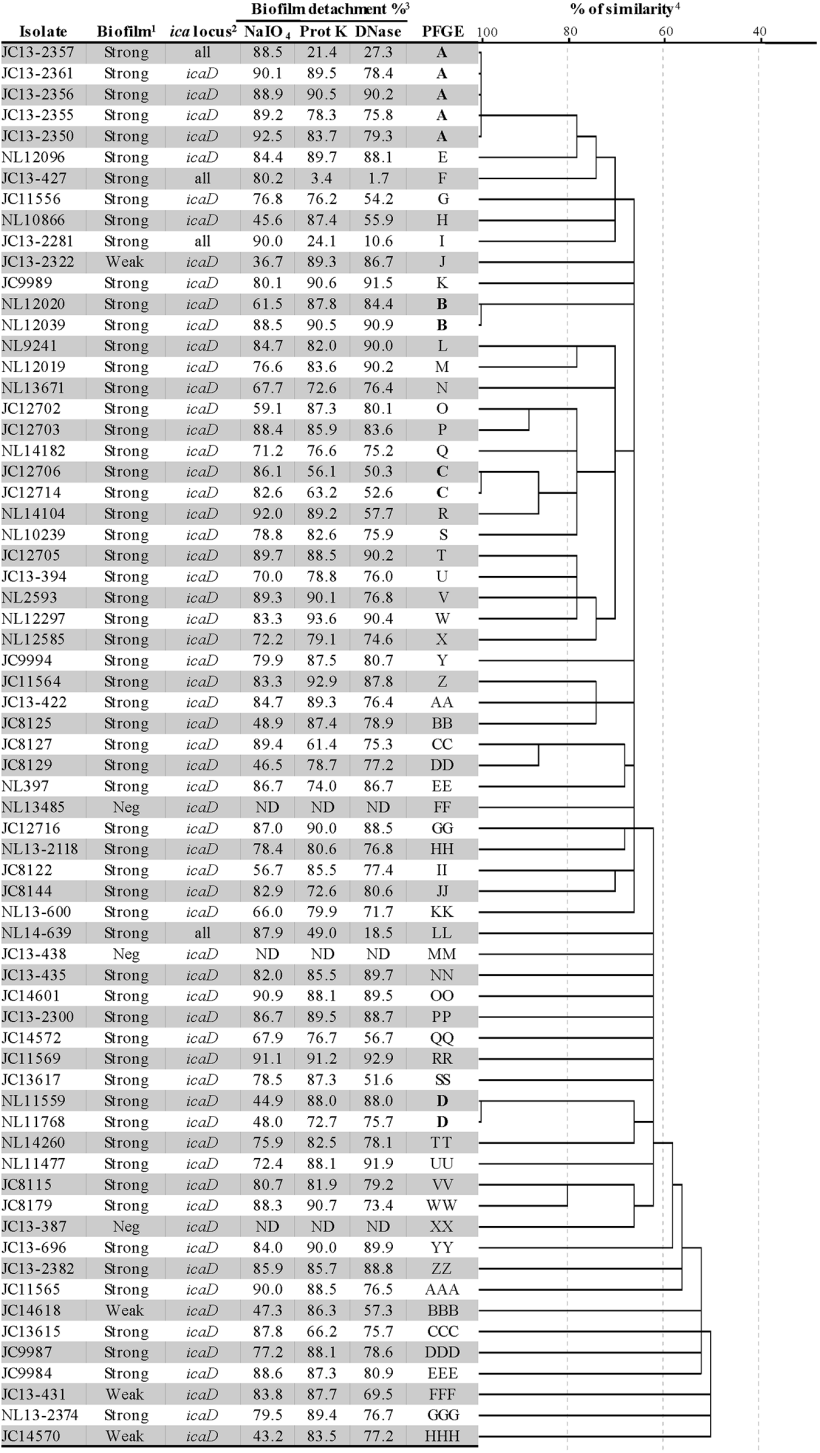
Pulsed-field gel electrophoresis dendrogram and biofilm production of *S*. *hominis* isolates. ^1^Biofilm production level: OD <0.12 negative, 0.12–0.24 weak and >0.25 strong. Neg: negative. ^2^All: positive for *icaR*, *icaA*, *icaD*, *icaB*, and *icaC*. ^3^ND non-determinate. ^4^Similarity coefficients were generated from a similarity matrix calculated with the Jaccard coefficient using SPSS 22.0 software.

Pulsed-field gel electrophoresis of *S*. *hominis* isolates revealed 62 different restriction patterns that had at least three band differences between each pattern. Four clones were detected (Fig 1). Clone A was represented by five isolates that were all recovered in November 2013 from the paediatric intensive care unit of Hospital Civil in Guadalajara, Mexico. Two isolates represented clones B, C, and D; each clone was isolated in the same month and from the same area and hospital.

### Biofilm formation and *icaADBC* operon

**Fig 2:**
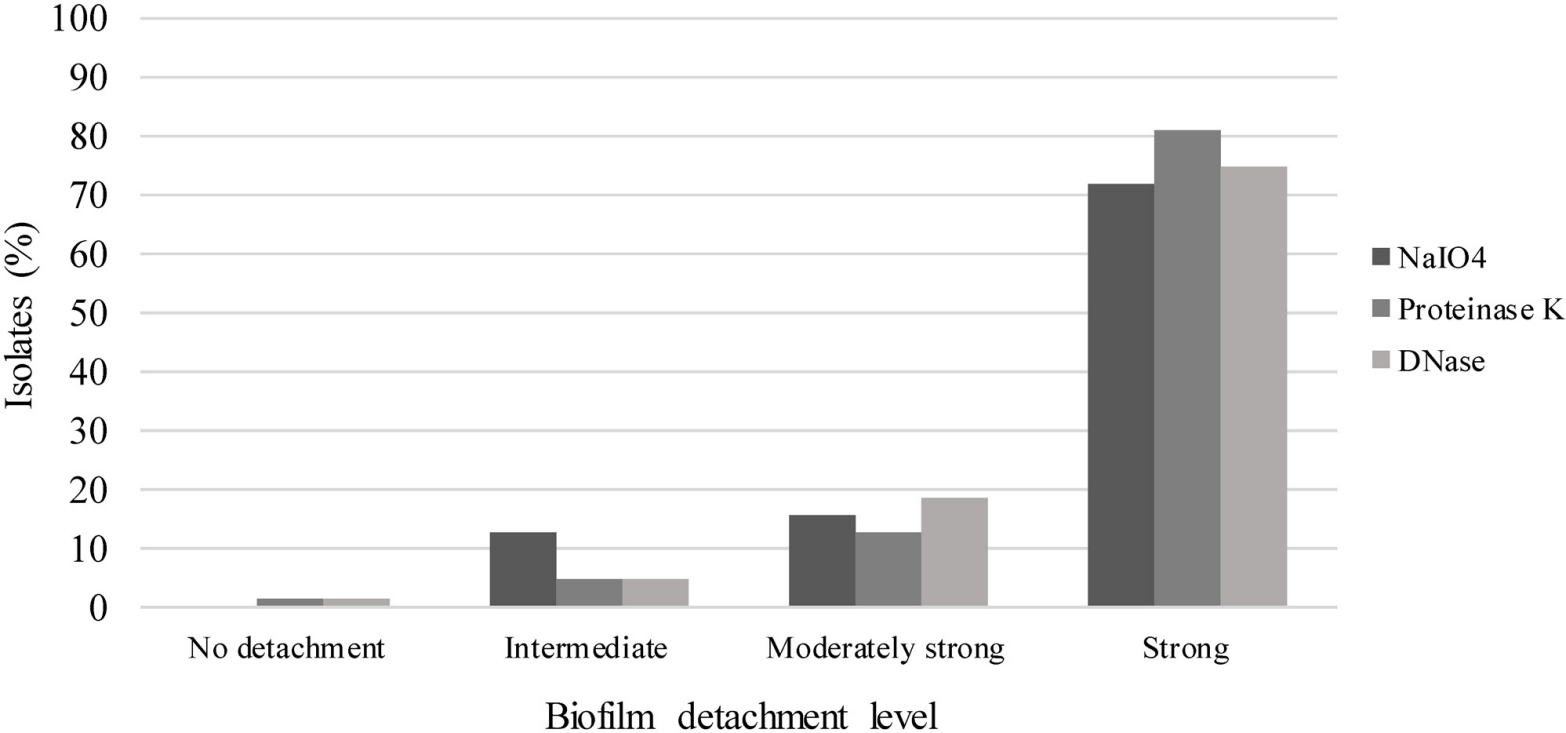
Biofilm detachment with NaIO4, proteinase K and DNase. Biofilm detachment level of 64 biofilm producers *S*. *hominis* isolates after treatment with NaIO_4_, proteinase K and DNase.

Of the 67 *S*. *hominis* isolates, 91% were categorised as strong biofilm producers as defined by the cut-off values proposed by Christensen *et al*.[18] Five isolates (7.5%) were identified as weak biofilm producers and three isolates (1.5%) as non-producers. The average BI values were 0.181, 0.360, and 2.542 for non-weak, and strong biofilm production, respectively. NaIO_4_, proteinase K and DNase showed a similar effect on reduction biofilm biomass (Figs 1 and 2). The *icaD* gene was detected in all isolates (100%) and four (7.5%) isolates harboured all five *ica* genes. Expression of *icaD* gene was performed in 15 strong biofilm producers *icaD* positive isolates, from which, only 1/15 (6.66%) isolate (NL14-639) expressed *icaD*. This isolate expressed *icaD* more than two times than *tuf* gene that was used as internal control for normalization and presented the five genes of *ica* operon. We did not find an association between clone type and biofilm production, as all clones were strong biofilm producers (Fig 1).

### MIC and MBEC

For almost all antibiotics tested, the resistance rate was significantly higher for biofilm cells than for planktonic cells (Table 1). The minimum concentrations that eradicated the biofilms of 50% and 90% of isolates (MBEC_50_ and MBEC_90_, respectively) were more than two-fold higher than the minimal concentrations that inhibited 50% and 90% of isolates in planktonic form (MIC_50_ and MIC_90_, respectively) for erythromycin, trimethoprim, amikacin, and vancomycin. This difference was also observed for oxacillin and ciprofloxacin, but only for MBEC_50_ compared to MIC_50_. For linezolid, the MBEC_90_ value was two-fold higher than the MIC_90_ value. We did not observe a significant difference between the MBEC and MIC values for chloramphenicol; however, the chloramphenicol resistance rate of biofilm cells was still two-fold higher than the resistance rate of planktonic cells. None of the 67 isolates tested in this study were resistant to vancomycin or linezolid as planktonic cells. However, 4.5% of isolates showed intermediate MIC values for vancomycin as biofilm cells, and 6% of the isolates were resistant to linezolid as biofilm cells.

**Table 1:** Antibiotic resistance of biofilm and planktonic cells of the isolates.

		(mg/L)^b^			
Antibiotic ^a^	Cells	MIC_50_ MBEC_50_	MIC_90_ MBEC_90_	%R (*n*)^c^	%S (*n*)^c^
ERY	Planktonic	256	512	77.6 (52)	23.4 (15)
	Biofilm	**> 1024**	**> 1024**	88.1 (59)	11.9 (8)
TMP	Planktonic	64	256	79.1 (53)	20.9 (14)
	Biofilm	**512**	**> 1024**	82.1 (55)	17.9 (12)
AMK	Planktonic	1	2	3 (2)	97 (65)
	Biofilm	**4**	**16**	9 (6)	91 (61)
VAN	Planktonic	0.5	1	0 (0)	100 (67)
	Biofilm	**2**	**4**	4.5 (3)*	95.5 (64)
LZD	Planktonic	1	1	0 (0)	100 (67)
	Biofilm	2	**4**	6 (4)	94 (63)
OXA	Planktonic	2	256	85 (57)	15 (10)
	Biofilm	**8**	512	92.5 (62)	7.5 (5)
CIP	Planktonic	8	64	58.2 (39)	41.8 (28)
	Biofilm	**32**	128	62.7 (42)	37.3 (25)
CHL	Planktonic	8	64	22.4 (15)	77.6 (52)
	Biofilm	8	128	**44.8** (30)	55.2 (37)

^a^ERY: erythromycin; TMP: trimethoprim; AMK: amikacin; VAN: vancomycin; LZD: linezolid; OXA: oxacillin; CIP: ciprofloxacin; CHL: chloramphenicol.

^b^MIC_50_ and MIC_90_: minimal concentrations that inhibit 50% and 90% of isolates, respectively (planktonic cells). MBEC_50_ and MBEC_90_: minimum concentrations that eradicate the biofilm of 50% and 90% of isolates, respectively. Values in bold indicate a significant difference in MICs and MBECs between planktonic and biofilm cells;

^c^ %R and %S: percentage of isolates resistant and susceptible, respectively. This classification was according to cut-offs of proposed by the CLSI.

*Value intermediate.

### Antibiotic susceptibility and biofilm index

**Fig 3:**
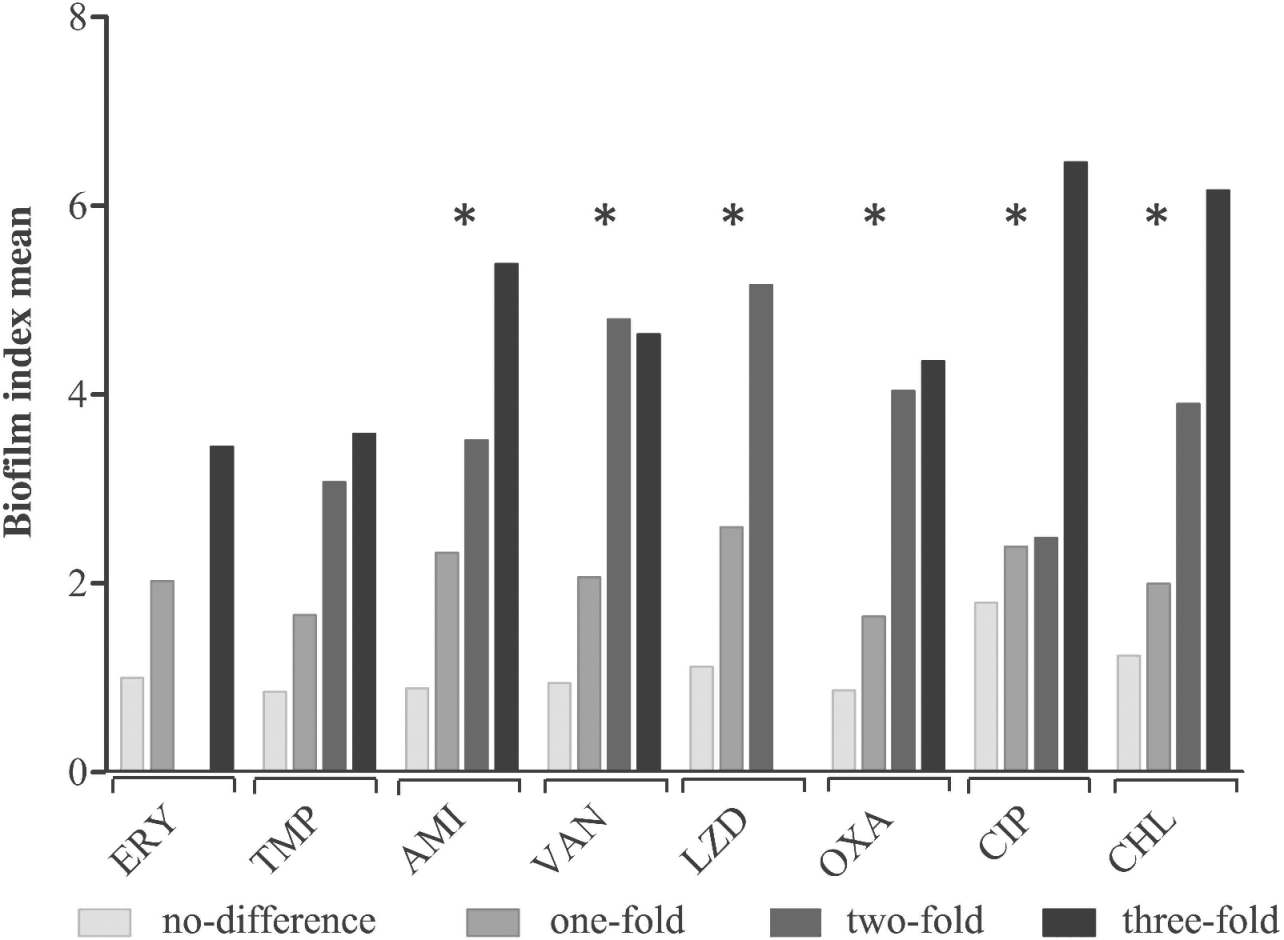
Increase BI mean and correlation with the differences in values observed between MIC and MBEC for all 67 isolates: no difference, one-fold increase, two-fold increase, three-fold increase. *Indicates a correlation between increases in antibiotic resistance (between MBEC and MIC) and BI mean. *p* < 0.05 by both ANOVA and Chi-square tests.

We observed higher BI and MBEC values compared to the MIC values for the following antibiotics: amikacin (p < 0.0001), vancomycin (p < 0.0001), linezolid (p < 0.0001), oxacillin (p < 0.0001), ciprofloxacin (p = 0.0005), and chloramphenicol (p < 0.0001) (Fig 3). This analysis was not determined for erythromycin and trimethoprim because several isolates had values above the upper limit of detection (>1024 mg/L) in both planktonic and biofilm cells.

Determinations at higher concentrations could not be performed because the antibiotic does not dilute at such concentration; therefore, we could not analyse the difference between MBEC and MIC values for these antibiotics.

## Discussion

Understanding the relative pathogenicity and virulence of *S*. *hominis* is crucial, in light of the recent increase antibiotic-resistant *S*. *hominis* infections, particularly those resistant to vancomycin and linezolid and those carrying the SCC*mec* gene.[10, 37] Recent phenotypic and molecular characterisations of *S*. *hominis* clinical isolates have found that *S*. *hominis* has low clonality, high methicillin resistance, and variable biofilm production. These studies have shown that *S*. *hominis* isolates frequently carry the *mecA* gene (likely as new SCC*mec* complex types), with a high prevalence of the *icaADBC* gene.[11–13, 22, 38] Herein, we have demonstrated that clinical isolates of *S*. *hominis* are less susceptible to antibiotics as biofilm cells.

Some studies have shown that production of biofilm in *S*. *hominis* is likely *ica*-independent, such as has been reported for *S*. *epidermidis*, *S*. *haemolyticus*, *S*. *aureus* and *S*. *lugdunensis*. [16, 22, 39–41]. The *S*. *hominis* isolates included in this study were strong biofilm producers, had a high frequency of the *icaD* gene and a low expression of this gene (6.66%). Has been proposed that *icaD* has co-expression with the *icaA* gene, which is responsible for polysaccharide synthesis by the production of *N*-acetylglucosamine oligomers and complete transferring the growing sugar chain to the cell surface. [35, 42] In this study, the *icaD* expression was demonstrated only in one isolate and NaIO_4_, proteinase K and DNase showed similar effect on reduction biofilm biomass (Fig 2). Therefore, the *N*-acetylglucosamine is not the major component, but one of the components of biofilm in *S*. *hominis*.

Biofilm production is an important virulence factor because biofilms facilitate bacterial adherence to biomedical surfaces (e.g. catheters, prosthetics, and cardiac valves) and entrance into the bloodstream.[19] Notably, this species has not previously been categorised as a major biofilm producer. Two previous studies reported that less than half of the *S*. *hominis* isolates were biofilm producers, or that the isolates were weak biofilm producers. However, these studies were performed on isolates obtained from surgical wounds, blood, catheters, or cerebrospinal fluid.[17, 22, 43] The discrepancy in the ability of these isolates to produce biofilm compared to the isolates we examined may be explained by the origin of the specimens used. Our strains were causative agents of laboratory-confirmed bloodstream infections and exclusively isolated from blood; therefore, these strains likely produced biofilm as a way to get into the bloodstream.

We observed differences in susceptibility between planktonic and biofilm cells for all antibiotics tested. Overall, isolates were more resistant to antibiotics as biofilms (Table 1). Cells may be more resistant to antibiotics as biofilms because they have reduced metabolic and growth rates (particularly cells deep within the biofilm), or because the biofilm matrix may adsorb or react with the antibiotics, thereby reducing the amount of antibiotics available to interact with cells in the biofilm. Another possibility is that the biofilm cells may have antibiotic tolerance. As a result of these factors, cells in the biofilm may be physiologically distinct from planktonic cells and, thus, express specific protective factors.[19, 20]

Antibiotic treatment protocols based on standard *in vitro* susceptibility tests designed for planktonic bacteria may fail to eradicate biofilm-producing *S*. *hominis* infections. This possibility is particularly concerning for monotherapies with vancomycin or linezolid, antibiotics to which *S*. *hominis* biofilms were remarkably resistant. Given these data, it may be more useful to base *S*. *hominis* treatment protocols on *in vitro* antibiotic susceptibility tests on biofilm cells. Our results are in agreement with reports on other CoNS species.[21] Caution should be taken before extrapolating these results to all CoNS species because of the high phenotypic and genetic variability in this species.

The BI value was associated with differences between the MBEC and MIC values. For example, with increasing BI values, we saw increasing differences between the MBEC and MIC values (Fig 3). This result suggests that the level of biofilm production may be proportional to the increase in antibiotic resistance. However, this possibility should be verified with more assays evaluating the biofilm structure and composition.

Planktonic cells were highly resistant to erythromycin, trimethoprim, oxacillin, and ciprofloxacin. Methicillin resistance and *mecA* gene frequency were also high (85%). Most isolates carried a non-typeable SCC*mec* complex, with a high percentage containing both *mec* complex A and *ccrAB1*. These results have been previously reported; however, it is important to continue monitoring the SCC*mec* complex in *S*. *hominis*, which often carries novel SCC*mec* types.[11–13] In this study, we detected a clone of 5 isolates that was strong biofilm producer, and isolates were collected from the paediatric intensive care unit. Several outbreaks of bloodstream infections among neonates and adults have been attributed to *S*. *hominis* subsp. *novobiosepticus*, which may account for the dissemination of these clones in the hospital environment. Additionally, *S*. *hominis* colonisation is frequently detected on the hands of nurses with skin lesions.[3, 44]

In conclusion, the *S*. *hominis* isolates analysed in this study were highly resistant to methicillin and other antimicrobials. Most of the SCC*mec* types detected were different from those described for *S*. *aureus*. We detected four clones, but in general, the isolates showed low clonality. The results of this study indicate that *S*. *hominis* is strong biofilm producer with an extracellular matrix with similar composition of proteins, DNA and *N*-acetylglucosamine. In addition, this species presents a high frequency of *icaD* gene and low expression of *icaD*. The biofilm production level is associated with antibiotic resistance.

## Supporting Information

S1 Table Characteristics and Results. (XLS)
